# Copper(II) Etioporphyrinate as a Promising Photoluminescent and Electroluminescent Temperature Sensor

**DOI:** 10.3390/ijms231810961

**Published:** 2022-09-19

**Authors:** Andrey Yu. Chernyadyev, Alexey E. Aleksandrov, Dmitry A. Lypenko, Aslan Yu. Tsivadze

**Affiliations:** A. N. Frumkin Institute of Physical Chemistry and Electrochemistry, Russian Academy of Sciences, Leninsky prosp. 31/4, 199071 Moscow, Russia

**Keywords:** etioporphyrin, tetrafluorenyl-porphyrin, copper, photoluminescence, electroluminescence, phosphorescence, thermosensors

## Abstract

Luminescent temperature sensors are of great interest because they allow remote determination of temperature in transparent media, such as living tissues, as well as on scattering or transparent surfaces of materials. This study analyzes the luminescent properties of copper(II) etioporphyrinate (Cu-EtioP) in a polystyrene film upon variation of temperature from −195 °C to +65 °C in a cryostat. It is shown that the ratio of intensities of phosphorescence transitions in the red spectral region of such a material varies significantly, that is, the material has thermosensory properties. The phosphorescence decay curves of copper(II) etioporphyrinate in a polystyrene film are analyzed. The quantum yield of phosphorescence of copper(II) etioporphyrinate determined by the absolute method was 3.15%. It was also found that the electroluminescence (EL) spectra of copper(II) etioporphyrinate in a poly(9-vinylcarbazole) (PVK) matrix demonstrated a similar change in the spectra in the temperature range −3 °C to +80 °C. That is, copper(II) etioporphyrinate can also be used as a luminescent temperature sensor as part of an active OLED layer.

## 1. Introduction

As is well-known, organic compounds belonging to the macroheterocycle class have found wide application due to a large set of properties that allow the use of phthalocyanines, porphyrins, and expanded porphyrins as materials for optical and electron-optical devices, e.g., photovoltaic converters, electroluminescent devices, and sensors for cations, anions, and triplet molecular oxygen [1,2,3,4]. Of particular interest in this class of compounds are metal porphyrinates with various peripheral substituents, which can significantly affect the optical properties of the porphyrin molecule in addition to the effect of the nature of the metal cation associated with the porphyrin cycle [2,4].

It was previously shown that metal porphyrinates with a closed electron shell are mainly characterized by a fluorescent type of luminescence, with the exception of porphyrinates containing metal cations that exhibit a significant ‘heavy atom’ effect (palladium, rhodium, ruthenium, platinum, osmium, iridium) [2,4]. Palladium and platinum porphyrinates were widely studied in the early 2000s as doping additives for electroluminescent devices based on light-emitting polymers [5,6]. Such doping additives, due to the ‘heavy atom’ effect and energy transfer via the Foerster or Dexter mechanism, made it possible to avoid the accumulation of the polymer triplet states during the operation of the electroluminescent device [5,6]. The possibility of using these compounds as luminescent temperature sensors was shown with the example of rhodium(III) and palladium(IV) crown porphyrinates, in which the ratio of the intensity of fluorescence and phosphorescence transitions changes with temperature [4,7]. Porphyrinates of metals with an open electron shell, such as vanadyl and copper(II) porphyrinates, are of particular interest from the point of view of luminescent thermosensors, since these compounds manifest significant changes in the phosphorescence lifetime with temperature [8,9]. We have previously shown that copper(II) tetrafluorenyl-porphyrinate can be considered as a luminescent thermosensor, since this complex has a large change in the phosphorescence lifetime with temperature changes as demonstrated by measurements of its phosphorescence lifetimes and phosphorescence decay curves [9]. However, the phosphorescence spectra of copper(II) tetrafluorenyl-porphyrinate changed very slightly with temperature [9].

In this study, we focused on copper(II) etioporphyrinate, in which the ratio of the phosphorescence intensity of the ^2^T_1_ trip-doublet and the ^4^T_1_ trip-quartet electron-excited states changes with temperature. The shape of the phosphorescence spectra changes very significantly with temperature as compared to other semi-synthetic porphyrinates of copper(II) and vanadyl (according to our data research). We analyzed changes in the photoluminescence spectra of copper(II) etioporphyrinate in a polystyrene film that protects copper(II) etioporphyrinate molecules from contact with triplet molecular oxygen molecules in a wide temperature range from −195 °C to +65 °C. We also analyzed changes in the electroluminescence spectra of copper(II) etioporphyrinate in the PVK matrix, since the mechanism of formation of triplet excited states of organic compounds and metal complexes in the electroluminescent process differs from photoexcitation and is characterized by different spin statistics [10,11]. Therefore, it was also of interest to identify differences in changes in the photo- and electroluminescence spectra of copper(II) etioporphyrinate with temperature.

## 2. Results

### 2.1. Synthesis of Copper(II) Etioporhyrinate (Cu-EtioP)

**Figure 1:**
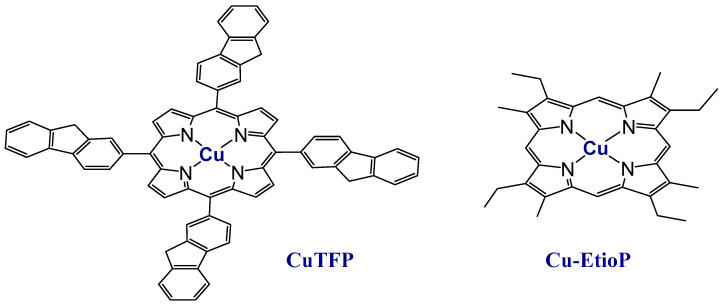
Structural formulas of copper(II) tetrafluorenyl-porphyrinate **CuTFP** and copper(II) etioporphyrinate **Cu-EtioP**.

Copper(II) etioporphyrinate (Figure 1) was obtained from copper(II) acetate and the free base of etioporphyrin I in a mixture of 96% ethanol/chloroform solvents while stirring the reaction solution for 30 min at 40 °C. This technique is widely used for the synthesis of copper(II) porphyrinates of various structures [12] provided that the free porphyrin base is well-soluble in mixtures of chloroform/ethanol or chloroform/methylene chloride. Benzonitrile can also be successfully used as a solvent [13]. Copper(II) etioporphyrin can be obtained under these conditions with a yield close to quantitative. The structure of the resulting compound was determined by MALDI-TOF mass spectrometry (without using any matrix), since the standard method of ^1^H NMR spectroscopy is not suitable for analyzing the structure of copper(II) porphyrinates due to their paramagnetism.

### 2.2. Photoluminescence of Cu-EtioP and Thermosensitivity of the Emission Bands

**Figure 2:**
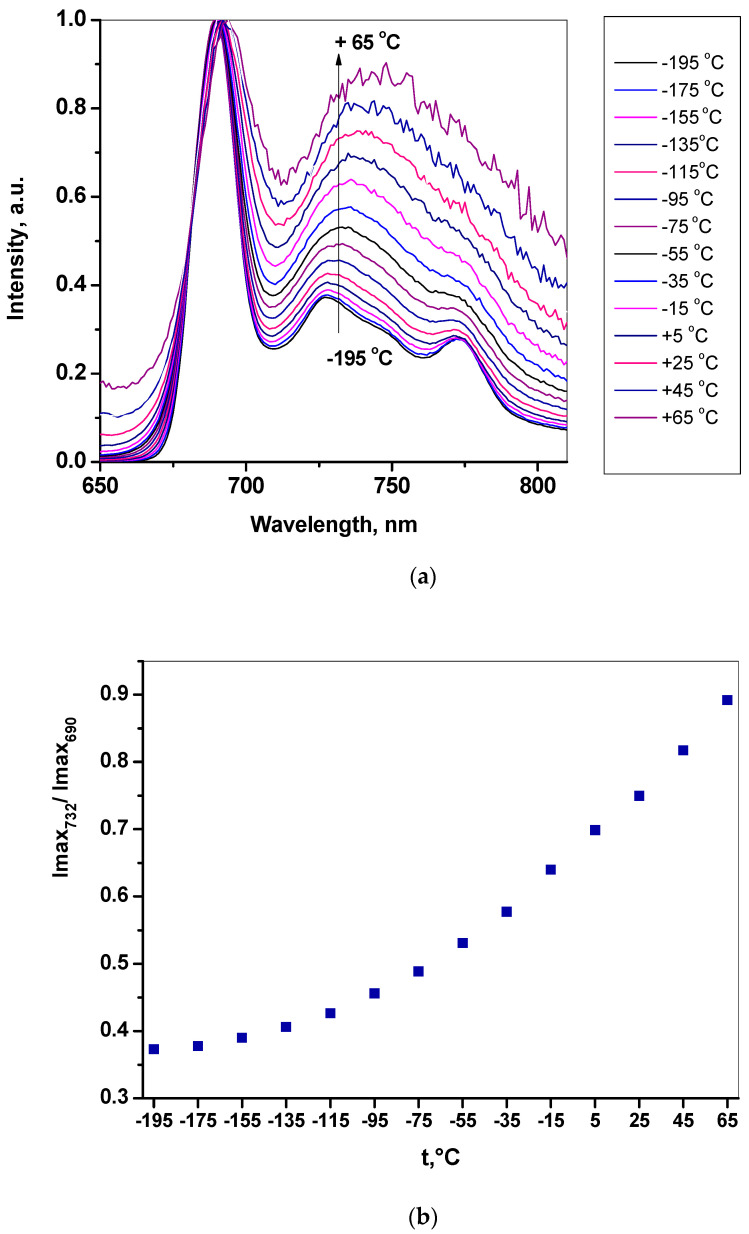
Temperature dependence of photoluminescence spectra for **Cu-EtioP** in polystyrene film. (**a**) The spectra measured using a Linkam cryostat with a 20 °C interval from −195 °C to 65 °C; (**b**) Temperature dependence of the PL intensity ratio (**I_732nm_/I_690nm_**) at band maxima.

The photoluminescent properties of copper(II) etioporphyrinate were studied in a polystyrene film to protect dye molecules from contact with triplet oxygen molecules in the air. In the photoluminescence spectrum of **Cu-EtioP** in a polystyrene film at 25 °C, two main maxima of emission bands are observed at 693 nm and 742 nm (Figure 2).

**Scheme 1:**
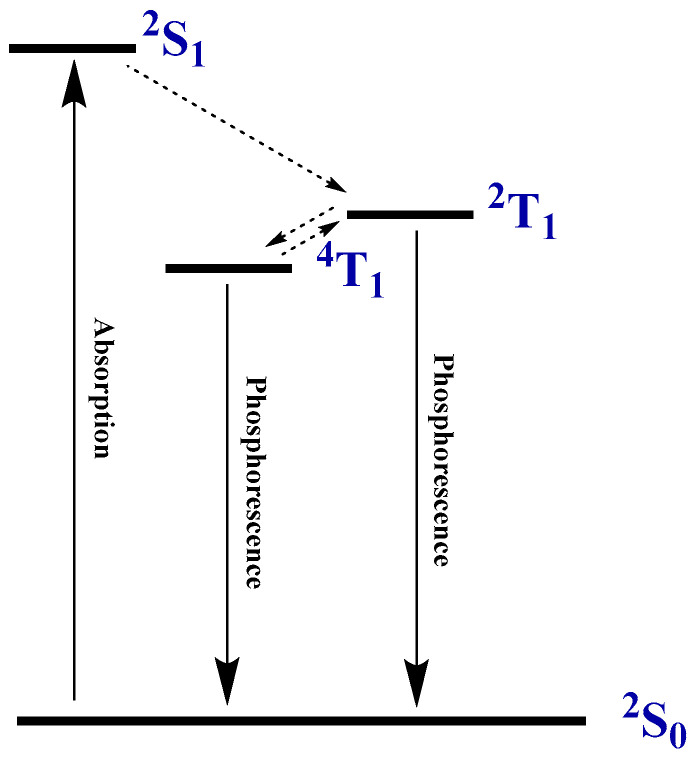
Photophysical processes in the molecules of copper(II) and vanadyl porphyrinates with open electron shells.

However, when the sample is cooled to −195 °C, the spectrum becomes better resolved and the ratio of the intensities of the main maxima changes significantly. This can be explained by the different temperature dependence of the rate constants of the radiative transitions from the trip-doublet ^2^T_1_ and trip-quartet ^4^T_1_ electronic levels to the main sing-doublet level ^2^S_0_ (Scheme 1).

**Figure 3:**
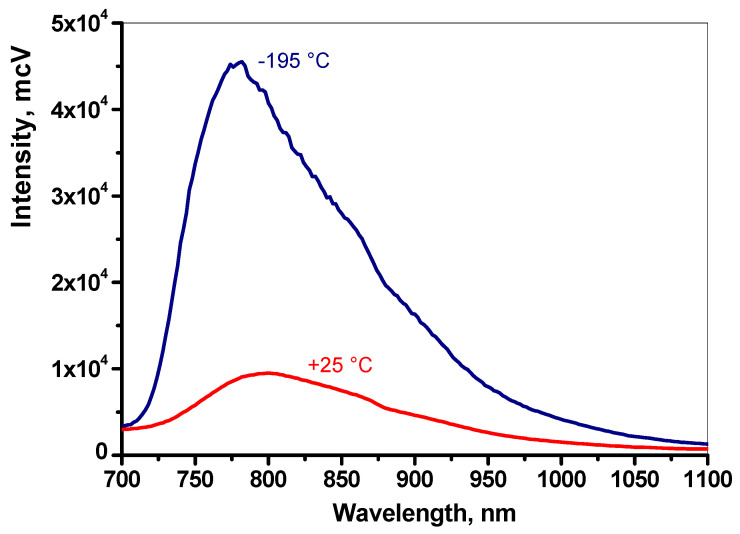
Photoluminescence spectra of copper(II) tetrafluorenyl-porphyrinate **CuTFP** in polystyrene film. Dark blue line: T = −195 °C; red line: T = 25 °C [9].

Such transitions, as already noted, occur in porphyrinates of metals with an open electron shell: vanadyl, copper(II), and chromium(III) [8,9]. However, not all such metal porphyrinates demonstrate such pronounced thermal sensitivity in their luminescence spectra as copper(II) etioporphyrinate. For example, copper(II) tetrafluorenyl-porphyrinate [9] and vanadyl tetraphenylporphyrinate [8] have weakly structured photoluminescence spectra at 25 °C and even at −196 °C (Figure 3). Luminescence in these complexes is realized only from the ^4^T_1_ level [8], while in copper(II) etioporphyrinate radiative transitions are realized from both levels ^4^T_1_ and ^2^T_1_. The emission band at 690 nm appears to correspond to the ^2^T_1_–^2^S_0_ transition of copper(II) etioporphyrinate, and in the 700–825 nm region, the observed luminescence spectrum is an overlap of the components of the emission band corresponding to the ^2^T1–^2^S_0_ transition and the weakly structured spectrum corresponding to the ^4^T_1_–^2^S_0_ transition.

We carried out a detailed analysis of the change in photoluminescence spectra for a sample of copper(II) etioporphyrinate in polystyrene in the temperature range from −195 °C to +65 °C in increments of 20 °C using a Linkam cryostat. This compound may be of interest as a luminescent temperature sensor in a manner analogous to the palladium(IV) crown porphyrinate we studied earlier, in which the ratio of the intensities of the fluorescence and phosphorescence lines changed with temperature [7]. At low temperatures, the line intensity ratio in the spectrum of CuTFP is 0.38 (I_732nm_/I_690nm_) at −195 °C and at first does not change much (Figure 2). However, as the temperature increases, the changes in the spectra become more significant and, at a temperature of 65 °C, the value of I_732nm_/I_690nm_ is already 0.87. This is perhaps due to the increasing role of the energy exchange between the trip-doublet ^2^T_1_ and trip-quartet ^4^T_1_ electronic levels with temperature, as the latter has lower energy and becomes more populated with an increase in temperature (Scheme 1). Emission transitions in the **Cu-EtioP** photoluminescence spectrum show a slight red-shift with increasing temperature, which is explained by the Stokes shift of spectral lines with an increase in the population of vibrational satellites of the electron levels of the molecule with temperature [14,15].

**Figure 4:**
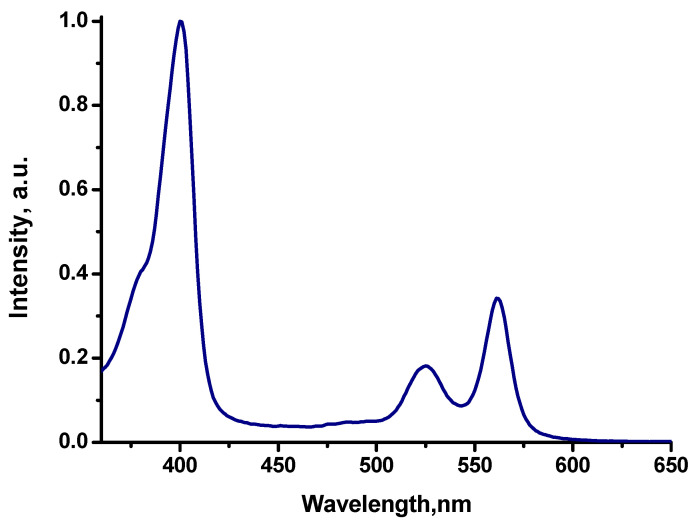
Photoluminescence excitation spectrum of **Cu-EtioP** in polystyrene film at 25 °C.

Photoluminescence-excitation spectra of **Cu-EtioP** in a polystyrene film (Figure 4), measured at different wavelengths of 690, 728, and 773 nm, coincide. The position of the maxima of the Soret and Q bands, as well as the ratio of their intensities, remain unchanged in the luminescence-excitation spectra measured at these three wavelengths. Therefore, the intensity ratio of the lines in the Cu-EtioP photoluminescence spectrum will remain unchanged upon a possible change in the wavelength of the exciting light, which seems important for the use of luminescent thermosensors [7]. In comparison with copper(II) tetrafluorenyl-porphyrinate **CuTFP** (Figure 1), copper(II) etioporphyrinate has a slightly lower photoluminescence quantum yield and an insignificant phosphorescence-lifetime change from 45 microseconds at −195 °C to 6 microseconds at 25 °C (See Table 1).

### 2.3. Electroluminescence of Cu-EtioP in PVK Matrix and Thermosensitivity of the Emission Bands

**Figure 5:**
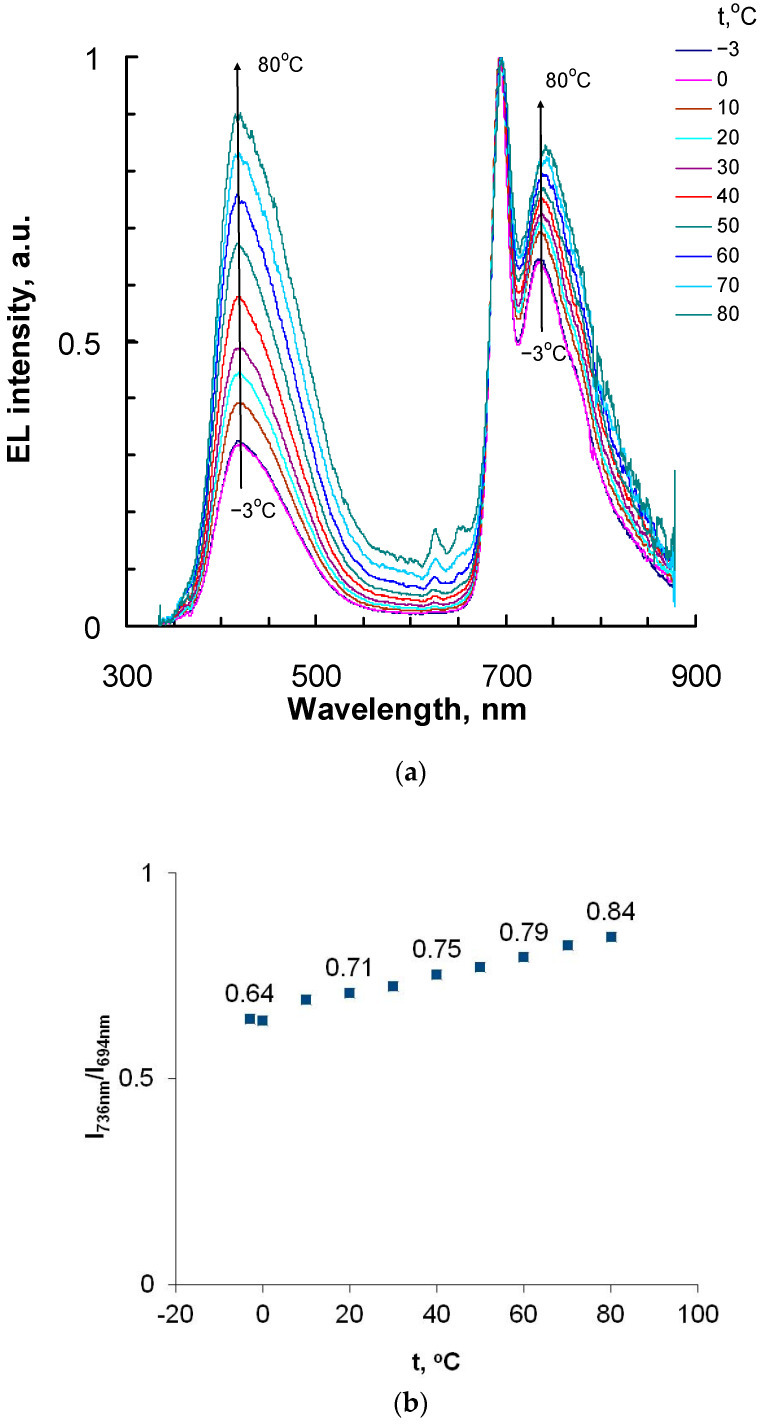
(**a**) Normalized EL spectra of OLED with Cu-EtioP content of 1 wt% in the light-emitting layer at temperatures from −3 °C to 80 °C at an applied voltage of 10 V. (**b**) Temperature dependence of the EL intensity ratio (**I_736nm_/I_694nm_**) at band maxima 736 and 694 nm.

Since the spin statistics in the process of photo- and electroexcitation of molecules of organic compounds and metal complexes can differ significantly, for example, 50%/50% between singlet and triplet excited states in the process of photoexcitation of molecules with a closed electron shell and ~25%/75% between singlet and triplet excited states in the process of electroexcitation for the same molecules [10,11], we analyzed the electroluminescence spectra of **Cu-EtioP** in the PVK matrix at a changing temperature (Figure 5) and compared them with the photoluminescence spectra of **Cu-EtioP** (Figure 2).

**Figure 6:**
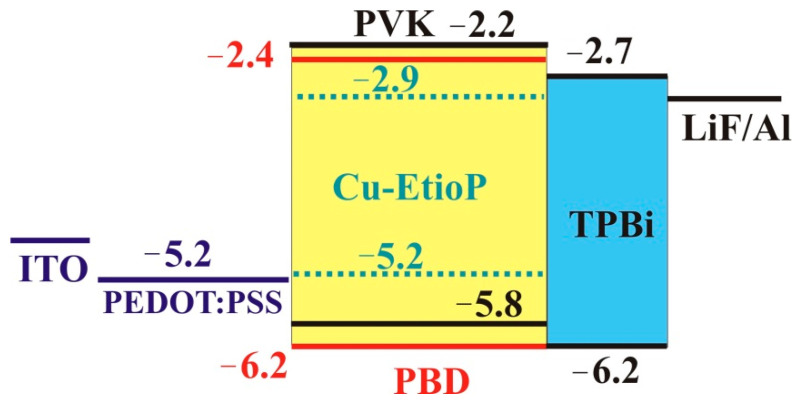
Energy diagram (eV) for the OLED studied with **Cu-EtioP** in the light-emitting layer.

To determine the dependence of the shape of the electroluminescence spectrum on temperature, OLEDs of the following structure were manufactured: ITO/PEDOT:PSS (30 nm)/PVK:PBD:**Cu-EtioP** (70 nm)/TPBi (20 nm)/LiF(1 nm)/Al (80 nm), where PEDOT:PSS is a hole-injection layer and TPBi [16] is an electron-transporting material (blocking holes). The light-emitting layer consisted of a mixture of PVK:PBD and **Cu-EtioP** with a mass component ratio of 70:30:1. With a higher **Cu-EtioP** content in the composite, concentration quenching occurs and the intensity of the EL decreases at the same applied voltage. PVK:PBD composite is used to create OLEDs formed by spin-coating from solutions and includes a wide range of phosphors, such as, for example, heavy metal complexes with ligands of different chemical nature [17,18,19] which have phosphorescent properties. PVK serves as a wide-band film-forming matrix with a high position of triplet levels [20], and PBD is an electron-conductive dopant which ensures the balance of charge carriers in the light-emitting layer. The energy levels of **Cu-EtioP** measured using cyclovoltammetry (CVA), according to the method described in [13,21], are well-suited for the PVK:PBD composite. The OLED energy diagram is shown in Figure 6.

For the OLED, EL spectra were recorded (Figure 5) at various temperatures at an applied voltage of 10 V. To do this, the sample placed on the Peltier elements was first cooled to −3 °C, and then heated to 80 °C. Three bands with maxima at 417, 694 and 736 nm are present in the EL spectra (Figure 6). Bands with maxima at 694 and 736 nm belong to **Cu-EtioP**, and the band in the blue region of the spectrum (λ_max_ = 417 nm) belongs to the PVK:PBD composite [17].

**Figure 7:**
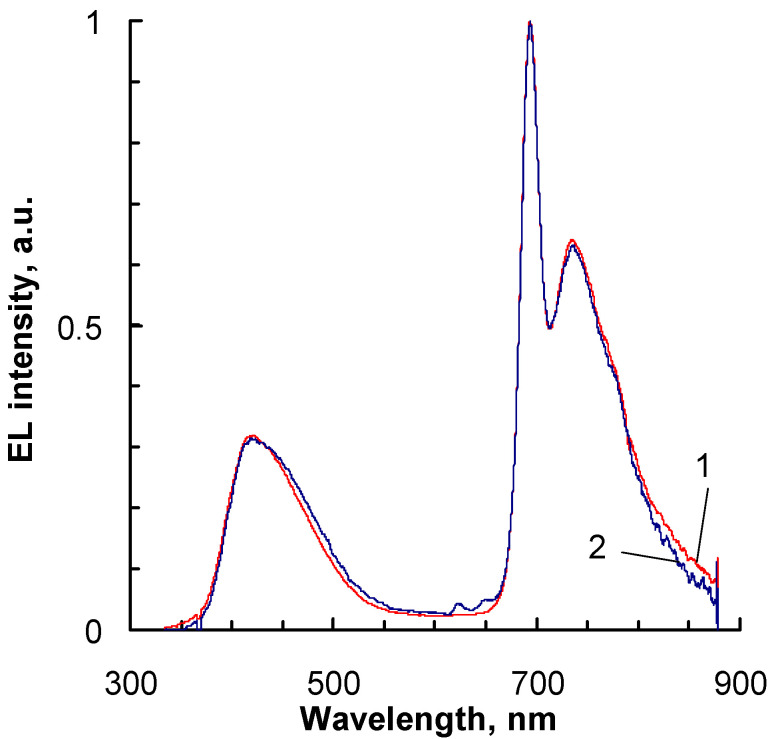
EL spectra at a temperature of 0 °C: (1) before heating; (2) after cooling from 80 °C.

With increasing temperature, an increase in the intensity of the band at 736 nm relative to the band at 694 nm is observed from I_736nm_/I_694nm_ = 0.64 at −3 °C to 0.85 at 80 °C, which closely corresponds to changes in the photoluminescence spectra in the same temperature range. The visible increase in the intensity of the band in the blue region of the spectrum (λ_max_ = 417 nm) in Figure 5 is due to the fact that the intensity of the bands of the **Cu-EtioP** phosphorescent compound (λ_max_ = 694, 736 nm) decreases markedly with increasing temperature, while the intensity of the band belonging to the PVK:PBD composite (λ_max_ = 417 nm) remains almost unchanged in a wide temperature range of 10–50 °C (Figure S1). The overall decrease in the intensity of OLED radiation at temperatures above 50 °C is apparently due to the degradation of the TPBi electron-transporting layer deposited by thermal vacuum evaporation. When the temperature drops to 0 °C after heating to 80 °C, the EL intensity of the OLED decreases, but the ratio of the intensity of the bands with maxima at 694 and 736 nm remains the same as before heating (Figure 7), which indicates the reversibility of photophysical processes occurring in **Cu-EtioP** molecules.

**Figure 8:**
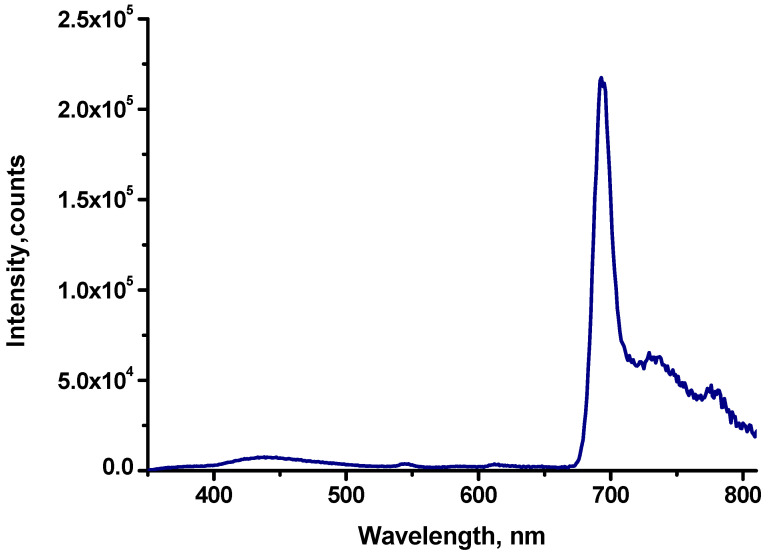
EL spectra of EL OLED with Cu-EtioP at −195 °C (dark blue line) measured at an applied voltage of 14 V in Dewar of a Horiba Flurolog 3 instrument.

Since the sealed chamber of the Linkam cryostat does not allow placing in it an OLED with wires from a power source, we were unable to conduct a detailed analysis of the spectra at negative temperatures. However, we placed the OLED in a Horiba Dewar vessel with a transparent lower part and measured the phosphorescence spectra of the OLED when cooled to −195 °C (Figure 8). In the electroluminescence spectrum (Figure 8) measured at a voltage of 14 V, a low-intensity matrix emission band in the region of 400–500 nm and an intense **Cu-EtioP** spectrum are observed. The intensity ratio I_732nm_/I_690nm_ = 0.31; it is also possible to note a close coincidence of the shape of the electroluminescence and photoluminescence spectra of **Cu-EtioP** at a temperature of −195 °C.

As can be seen, the electroluminescence spectra of **Cu-EtioP** are very similar in shape to the photoluminescence spectra of **Cu-EtioP** both at room temperature and at −195 °C (Figure 2, Figure 5, and Figure 8, respectively). In the temperature range from −3 °C to +80 °C, the nature of changes in the electroluminescence spectra of **Cu-EtioP** is similar to changes in the photoluminescence spectra of **Cu-EtioP**. Such a close analogy between the photo- and electroluminescence spectra allows us to conclude that the channels of radiative transitions in both processes are virtually identical. In the processes of photo- and electroexcitation of the **Cu-EtioP** molecule, emission transitions are realized from the triplet levels ^2^T_1_ and ^4^T_1_ of the **Cu-EtioP** molecule; emission from the singlet-doublet level ^2^S_1_ is completely absent in the photoluminescent process (Scheme 1, Figure 2, Figure 5 and Figure 8).

## 3. Discussion

In the positive temperature range, the sensitivity of Cu-EtioP luminescence to temperature changes is comparable to the palladium(IV) porphyrinate we studied earlier [7]. The ratio of phosphorescence intensity to fluorescence in palladium crown porphyrinate varies by 32% with a temperature change from 25 to 75 °C, if intensity of phosphorescence/intensity of fluorescence at 25 °C is taken as 100%. The ratio of the intensity of phosphorescence transitions with a maximum emission at 732 nm and at 690 nm in copper(II) etioporphyrinate varies by 28% with a temperature change from 5 to 65 °C in the photoluminescence spectra. The ratio of the intensity of phosphorescence transitions with a maximum emission at 736 nm and at 694 nm for copper(II) etioporphyrinate changes by 31% with a temperature change from 0 to 80 °C in the OLED electroluminescence spectra.

In the negative temperature range, the sensitivity of Cu-EtioP luminescence to temperature changes is lower than that of palladium(IV) porphyrinate. The ratio of intensity of phosphorescence to fluorescence in palladium(IV) porphyrinate varies by 8.87 times when the sample is cooled from 25 to −196 °C. The ratio of the intensity of phosphorescence transitions with a maximum emission at 732 nm and at 690 nm in the photoluminescence spectra for copper(II) etioporphyrinate in polystyrene changes by 2.01 times when the sample is cooled from 25 to −195 °C. The ratio of the intensity of phosphorescence transitions with a maximum emission at 736 nm and at 694 nm for copper(II) etioporphyrinate changes 2.29 times when the sample is cooled from 25 to −195 °C in the electroluminescence spectra of the OLED. However, the accuracy of temperature measurements in the case of using Cu-EtioP as a sensor may be higher compared to palladium(IV) and rhodium(III) porphyrinate [3,7], since Cu-EtioP strongly changes the shape of the spectrum in the wavelength range 700–825 nm, and the entire shape of the spectrum can be used to determine temperature as opposed to only the ratio of intensities in the transition maxima emissions.

Currently, quite a lot of luminescent temperature sensors based on lanthanides have been created, for which the analysis of temperature changes is based on changes in the lifetime of phosphorescence [22,23]. However, such sensors require the use of relatively complex devices, such as a phosphorimeter or a photon-counting system, to register the phosphorescence or fluorescence decay curves. In addition, the luminescence intensity of the sensor must be relatively high to register a qualitative decay curve. Luminescent sensors, whose operation is based on measuring the intensity ratio of the emission bands, have clear advantages in terms of using a conventional fluorimeter to register spectra, and modern photomultiplier tubes and semiconductor detectors have very high sensitivity, so even a sensor with high-intensity luminescent glow is not required.

## 4. Materials and Methods

### 4.1. Synthesis of Copper(II) Etioporphyrinate Cu-EtioP

The “Sigma Aldrich” free base of etioporphyrin I was used in this work. First, 12 mg of the free base of etioporphyrin I were dissolved in 20 mL of CH_2_Cl_2_. Then, 7 mg of copper(II) acetate were dissolved in 15 mL of ethanol. The solutions were mixed and the resulting solution was stirred at 40 °C for 30 min. The solvents were removed in vacuo, the product was dissolved in CH_2_Cl_2_ and purified by column chromatography of traces of unreacted etioporphyrin I. The yield of copper(II) etioporphyrinate **Cu-EtioP** was 13 mg (96%). The structure of the compound obtained was confirmed by MALDI-TOF mass-spectrometry (m/z, I%: 539.2(100%), 541.2(44.8%), 540.2(34.5%)) and UV/vis spectroscopy in CH_2_Cl_2_ solution (λ_max_, nm: 401(Soret Band), 526,563(Q-Bands)).

### 4.2. Preparation of Cu-EtioP in Polystyrene Film

0.2 milliliters of 1 × 10^−6^ M solution of copper(II) etioporphyrinate **Cu-EtioP** in CH_2_Cl_2_ were added to a solution of 0.05 g of polystyrene (Aldrich, St. Louis, MO, USA) in 1 mL of toluene. The solution obtained was placed in a flat-bottom aluminum cup and left for 24 h in a fume hood. The obtained film was additionally dried in vacuo. A disc-like flat film with a 0.7 cm diameter was cut to the required size in order to place it in a Linkam cryostat.

### 4.3. Fabrication and Characterization of Organic Light-Emitting Diodes (OLEDs)

OLEDs based on the Cu-EtioP:PVK:PBD composites were manufactured according to the following procedure. ITO/glass substrates (Kintec) were thoroughly cleaned and treated with O_2_ plasma. A PEDOT:PSS (CLEVIOS P VP AI 4083) layer was deposited onto the ITO/glass substrate from an aqueous solution by spin-coating at 2000 rpm in air and then dried in an Ar atmosphere at 110 °C for 20 min. The light-emitting films of the ternary blends were prepared from the chlorobenzene solutions by spin-coating at 2000 rpm and dried at 80 °C for 1 h. The TPBi layer was deposited at a rate of 0.6 Å/s in vacuum of 2 × 10^−6^ mbar. The fabricated OLEDs were of the architecture ITO/PEDOT:PSS (30 nm)/PVK:PBD:Cu-EtioP(70 nm)/TPBi (20 nm)/LiF (1 nm)/Al (80 nm), where poly(3,4-ethylenedioxythiophene):poly- (styrenesulfonate) (PEDOT:PSS) was used as a hole-injection and transporting layer, PVK served as a hole-transporting material, 2-(4-biphenyl)-5-(4-tbutyl-phenyl)-1,3,4-oxadiazole (PBD) was an electron-transporting material doped with a **Cu-EtioP** compound, all the above were utilized as an emissive layer, and TPBi (2,2′,2′-(1,3,5-benzinetriyl)-tris(1-phenyl-1-H-benzimidazole)) was used as an electron transporting layer. Electroluminescence spectra of the OLEDs were recorded using a FL-3000 Fiber-Optics adaptor connected with a Fluorolog-3 instrument (HORIBA Jobin Yvon S.A.S., Edison, NJ, USA). The temperature dependence of the EL spectra was recorded using Peltier elements in an Ar atmosphere with O_2_ and H_2_O contents < 10 ppm. Electroluminescence (EL) spectra of the OLEDs at 77 K were recorded using a Dewar from HORIBA Jobin Yvon S.A.S. and Fluorolog-3 spectrofluorometer (HORIBA Jobin Yvon S.A.S.).

The photoluminescence spectra were recorded on the Fluorolog-3 spectrofluorometer (HORIBA Jobin Yvon S.A.S.). The excitation source was a 450 W xenon lamp with Czerny–Turner double monochromators; the registration channel was equipped by a R928 photomultiplier with Czerny–Turner double monochromators. A 150 W pulsed xenon lamp was used for measurements of phosphorescence decay curves. The phosphorescence decay curves were analyzed using the FluoroEssence software (HORIBA Jobin Yvon S.A.S.) for the calculation of the phosphorescence lifetime values. The quantum yield of **Cu-EtioP** photoluminescence in a polystyrene film was measured by the absolute method. The photoluminescence light was collected by a Quanta-φ F-3029- sphere connected to Fluorolog 3 by a FL-3000 Fiber-Optics adaptor (Horiba Jobin Yvon S.A.S). The FL-3000 Fiber-Optics adaptor was used both for excitation of **Cu-EtioP** photoluminescence in polystyrene film placed in a Linkam cryostat and for recording of photoluminescence spectra.

## 5. Conclusions

As a result of the study, it was found that copper(II) etioporphyrinate Cu-EtioP in polystyrene film demonstrates the thermal sensitivity of phosphorescence spectra. In the phosphorescence spectra, the ratio of the emission band intensities at 690 nm and 728 nm changed over a wide temperature range. Phosphorescence transitions in Cu-EtioP molecules were characterized by luminescence-excitation spectra that coincided in shape, which makes it possible to use any arbitrarily selected wavelength to register the Cu-EtioP phosphorescence spectrum in polystyrene, while the ratio of the intensity of the emission spectrum transitions will remain unchanged. Hence, it can be concluded that copper(II) etioporphyrinate in a polystyrene film can be considered as a promising material for fluorescent thermosensors along with the palladium(IV) and rhodium(III) crown porphyrinates described earlier [4,7]. Analysis of the electroluminescence spectra of Cu-EtioP in PVK showed that the electroluminescence spectra of the composite change in a very similar way when compared to the photoluminescence spectra of copper(II) etioporphyrinate in polystyrene. The differences in the spin statistics of the processes of electro- and photoexcitation of copper(II) etioporphyrinate molecules do not actually manifest themselves in the temperature dependence of the photo- and electroluminescence spectra. It is obvious that the Cu-EtioP compound can also be considered as an active component of electroluminescent thermosensors with operation based on a change in the intensity ratio of emission bands in the electroluminescence spectrum.

## Figures and Tables

**Table 1:** Photophysical data for copper(II) porphyrinates measured in polystyrene film.

Compound	Emission Peak, λ_max_, nm	QY, %	Life Time, µs
**Cu-EtioP**	693, 742 (T = 25 °C)	3.15 (T = 25 °C)	6 ** (T = 25 °C)
	690, 728, 773 (T = −195 °C)		45 (T = −195 °C)
CuTFP*	798 (T = 25 °C)	5.2 (T = 25 °C)	5 ** (T = 25 °C)
	779 (T = −195 °C)		344 (T = −195 °C)

* Data from [9] for **CuTFP**. ** Short life times of 5 or 6 microseconds cannot be measured accurately by a phosphorimeter with a xenon flashing lamp, therefore it can in fact be shorter than 5 or 6 microseconds.

## Supplementary Materials

The supporting information can be downloaded at: https://www.mdpi.com/article/10.3390/ijms231810961/s1.
