# Supplementary figures and images for: Copper(II) Etioporphyrinate as a Promising Photoluminescent and Electroluminescent Temperature Sensor
Source: Int J Mol Sci. 2022 Sep 19;23(18):10961. doi: 10.3390/ijms231810961 (PMC9504643; doi:10.3390/ijms231810961)

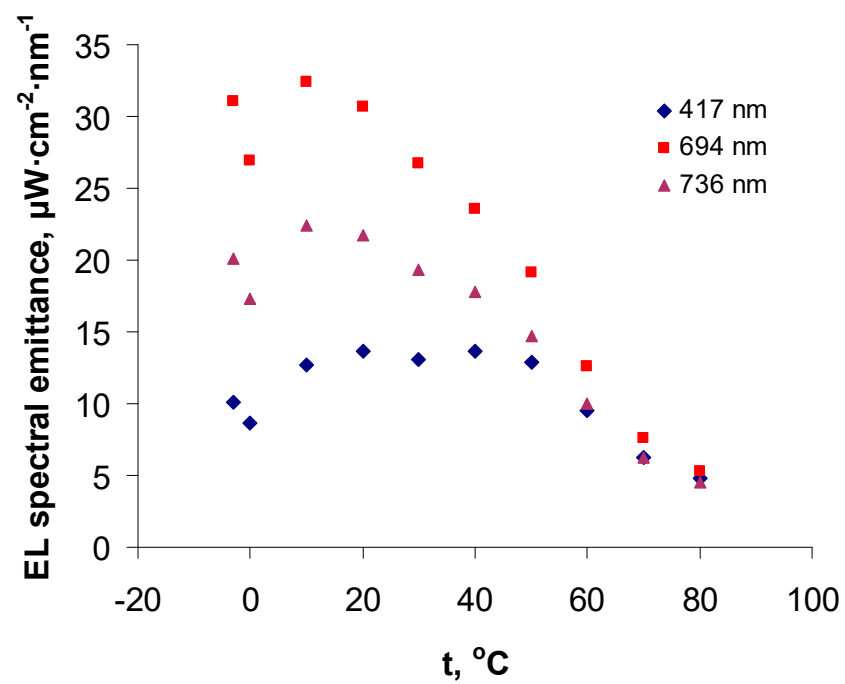

Figure S1. Temperature dependence of EL intensity at band maxima 417, 694 и 736 nm.

Supplement: Supplementary file 1 [file ijms-23-10961-s001.zip › ijms-1902730-supplementary.pdf]
